# Supplementary material for: On-Site Dual Detection of Airborne Acinetobacter baumannii and Its Carbapenem-Resistant Gene blaOXA-23 Using a One-Pot Visual LAMP-CRISPR/Cas12a-Based Platform
Source: Microorganisms. 2025 Apr 24;13(5):976. doi: 10.3390/microorganisms13050976 (PMC12114085; doi:10.3390/microorganisms13050976)
Supplement: Supplementary file 1 [file microorganisms-13-00976-s001.zip › microorganisms-3544228-supplementary.pdf]

supplementary information for

On-Site Dual Detection of Airborne *Acinetobacter baumannii* and Its Carbapenem-Resistant Gene *bla*$_{OXA-23}$ Using a One-Pot Visual LAMP-CRISPR/Cas12a-Based Platform

1. Supplementary methods

1.1 Bacteria culture and genomic DNA preparation

Acinetobacter baumannii (*A. baumannii*) was cultured in Trypticase Soy Broth (TSB; Hopebio, China) medium at 37 °C for 24 - 48 h with continuous shaking at 200 rpm. Other bacteria strains isolated from clinical samples were cultured on blood agar plates (Hopebio, China) at 37 °C for 24 - 48 h in a tertiary care hospital. Genomic DNA was extracted from all bacterial culture using the TIANamp Bacteria DNA Kit (Tiangen Biotech, China). The concentration of the extracted DNA was quantified using the Qubit dsDNA BR Assay kit (Thermo Fisher Scientific, USA). These DNA samples were subsequently used as templates for the establishment of detection assays.

1.2 crRNA design, preparation, and selection

crRNA design: For the design of crRNAs targeting *A. baumannii* and the *bla*$_{\text{OXA-23}}$ gene, the presence of a 5'-TTTV PAM sequence was required, and a spacer sequence of 20-23 nucleotides (nt) was preferred. To target *A. baumannii*, the nucleotides 'TA', 'TG', or 'TC' were introduced into the linker region of the BIP primer to generate a 5'-TTTV PAM sequence. For the *bla*$_{OXA-23}$ gene, the 5'-TTTV PAM sequences were directly found within the amplified target region. The secondary

structure of the crRNA, comprising the spacer sequence and the handle sequence, was predicted using UNAFold (https://www.unafold.org/).

crRNA preparation: The crRNA template was generated by annealing the forward primer (5'-GCGCTAATACGACTCACTATAGG(G)TAATTTCTACTAAGTGTAGAT-3') and the reverse primer (5'-spacer sequence complementary + ATCTACACTTAGTAGAAATTACC(C)-3'), followed by PCR amplification. The resulting PCR product was purified using an ethanol precipitation method. Then, the purified PCR products were subjected to in vitro transcription using the HiScribe T7 High Yield RNA Synthesis Kit (New England Biolabs, USA) at 37 °C for 16 h to generate crRNAs. The transcribed crRNAs were purified using the Monarch RNA Cleanup Kit (New England Biolabs, USA) according to the manufacturer's instructions. The concentration of the purified crRNAs was quantified using the Qubit Flex Fluorometer with the RNA High Sensitivity (HS) Assay Kit (Thermo Fisher Scientific, USA). Finally, the crRNAs were aliquoted and stored at -80 °C for subsequent experiments.

crRNA selection: The efficiency of the designed crRNAs was evaluated by the CRISPR/Cas12a-mediated cleavage assay. To select crRNAs targeting *A. baumannii*, four different LAMP primer sets were employed to amplify the target sequence. Following amplification, 25 μL of each LAMP amplicons was individually mixed with the CRISPR/Cas12a reaction mixture and incubated at 37 °C for 40 min to evaluate the cleavage efficiency of the designed crRNAs. In this step, the 25 μL CRISPR/Cas12a reaction mixture included 2X NEB buffer r2.1, 100 nM crRNA, 100 nM Lba Cas12a, 50 U of recombinant RNase inhibitor, 1 μM of reporter probe, and RNase-free water.

For the selection of crRNAs targeting the *bla*$_{OXA-23}$ gene, the F3 and B3 primers were used to

amplify the target sequence. Then, the PCR product served as the template to assess the functionality of the designed crRNAs. The Lba Cas12a cleavage-mediated assay was conducted in a total volume of 20 μL, including 1X NEB buffer r2.1, 50 nM crRNA, 50 nM Lba Cas12a, 20 U recombinant RNase inhibitor, 0.5 μM reporter probe, template (10^{10} copies) and RNase-free water.

The signal readout of the detection assay relied on the trans-cleavage activity of Lba Cas12a. Therefore, to further verify whether the CRISPR/Cas12a cleavage reaction is specifically activated by the target nucleic acid, thereby triggering its trans-cleavage activity, a reaction was performed using the target nucleic acid as the template. The CRISPR/Cas12a assay was conducted in a 20 μL reaction mixture containing 1× NEBuffer r2.1, 50 nM crRNA, 50 nM Lba Cas12a, 20 U recombinant RNase inhibitor, 2 μM FAM-labeled single-stranded DNA (ssDNA) reporter (FAM-TTTTTTTTATTATTT), approximately 10^{11} copies of the target nucleic acid, and RNase-free water. The mixture was incubated at 37 °C for 1 h, and the cleavage products were subsequently analyzed by 20% denaturing urea-PAGE at 220 V for 50 min.

1.3 Evaluation of mineral oil volume for LAMP reaction coverage

To evaluate the effect of mineral oil volume on evaporation prevention, 25 μL of food dye (as a substitute for the LAMP reaction mixture) was added to the bottom of reaction tubes, followed by the addition of varying volumes of mineral oil to form a protective layer. The tubes were then incubated in a heat block at 63 °C for 55 min. After incubation, the evaporation was observed visually.

1.4 Optimization of one-pot LAMP-CRISPR/Cas12a assay

The one-pot LAMP-CRISPR/Cas12a assay consisted of two components: the LAMP reaction and the CRISPR/Cas12a reaction. The LAMP reaction was performed as described in section 2.3 of the main text, using approximately 1 ng of template. To optimize the one-pot LAMP-CRISPR/Cas12a assay, the CRISPR/Cas12a reaction conditions were systematically adjusted, including the concentrations of Lba Cas12a, crRNA, and the reporter probe. All concentrations mentioned below represent the final concentrations in the 25 μL CRISPR reaction system.

For the optimization of Lba Cas12a concentration, a 25 μL CRISPR/Cas12a reaction mixture was prepared, containing 2X NEB buffer r2.1, 1:1 ratio of Lba Cas12a to crRNA at varying concentrations (60 nM:60 nM; 100 nM:100 nM; 200 nM:200 nM; 300 nM:300 nM), 50 U recombinant RNase inhibitor, 1 μM reporter probe, and RNase-free water.

For the optimization of crRNA concentration, a 25 μL CRISPR/Cas12a reaction mixture was prepared, containing 2X NEB buffer r2.1, 100 nM Cas12a, varying concentrations of crRNA (50 nM; 100 nM; 150 nM; 200 nM), 50 U recombinant RNase inhibitor, 1 μM reporter probe, and RNase-free water.

For the optimization of reporter probe concentration, a 25 μL CRISPR/Cas12a reaction mixture contained 2X NEB buffer r2.1, 100 nM Cas12a, 50 nM crRNA, 50 U recombinant RNase inhibitor, FAM or Rox reporter probe (0.2 μM, 0.4 μM, 1 μM, 2 μM) and RNase-free water.

1.5 Sanger sequencing analysis of five actual air samples

Nucleic acid extracted from the five air samples were amplified by PCR. The PCR reaction mixture contained template DNA, 1X Fast Pfu buffer with Mg^{2+}, 1 μM each of forward (F) and reverse (R) primer, 0.2 mM dNTPs, 1.25 U Fast Pfu DNA polymerase and nuclease-free water to a final volume of 50 μL. The sequence of *bla*$_{\text{OXA-23}}$-F primer and *bla*$_{\text{OXA-23}}$-R primers were CACTAGGAGAAGCCATGAAG and CAGCATTACCGAAACCAATACG, respectively Following amplification, the PCR products were analyzed by 1% agarose gel electrophoresis to verify their size. Products that matched the expected size were subsequently sent to Sangon Biotech Co., Ltd (Shanghai, China) for Sanger sequencing.

2. Supplementary results and figures

2.1 Results of crRNA selection

The results demonstrated that *Ab*-ITS-crRNA was able to cleavage the reporter probes in the system that contained the WT LAMP product, indicating its functional activity even in the absence of a canonical PAM sequence (Figure S1). Previous studies have also shown that Lba Cas12a-crRNA complex can recognize certain non-canonical PAM sequences [1]. Based on these findings, the WT LAMP primer set combined with *Ab*-ITS-crRNA was selected for the following experiment. For *bla*$_{OXA\text{-}23}$-crRNA selection, compared to other crRNAs, *bla*$_{\text{OXA-23}}$-crRNA1 showed the highest fluorescence signal (background subtracted fluorescence) among the tested crRNAs, demonstrating superior cleavage activity. So *bla*$_{\text{OXA-23}}$-crRNA1 was selected for further testing (Figure S1).

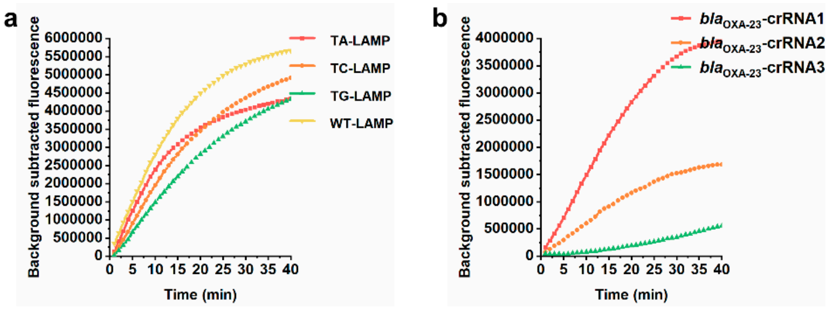

Figure S1. The results of Lba Cas12a-mediated cleavage reaction. a. The results of Lba Cas12a-mediated cleavage of different LAMP products in one-pot LAMP-CRISPR/Cas12a assay (Target: *A. baumannii*). TA-LAMP, TC-LAMP, and TG-LAMP referred to the LAMP reactions with the TA, TC, and TG sequences, respectively, which were artificially added to the BIP primer in the linker region between B1C and B2. WT-LAMP referred to no additional base added into BIP primer. b. The results of Lba Cas12a-mediated cleavage using three different crRNAs (Target: *bla*$_{OXA-23}$). Background subtracted fluorescence was defined as the fluorescence of the template-loaded reaction minus that of the corresponding reaction using RNase-free water as the template.

To further validate the trans-cleavage activity of the CRISPR/Cas12a cleavage reaction mediated by the selected crRNA in the presence of target dsDNA, we performed electrophoretic analysis of the cleavage products. As shown in Figure S2, ssDNA was effectively cleaved only in the presence of the target dsDNA, indicating that the CRISPR/Cas12a reaction mediated by the selected crRNA relies on trans-cleavage activity for the detection of target dsDNA.

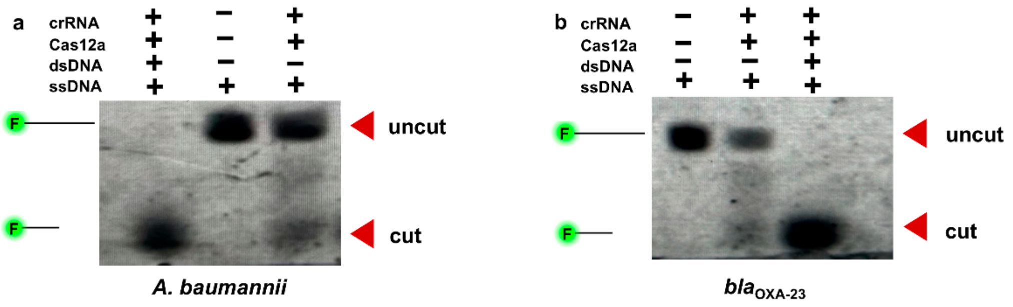

Figure S2. Urea-PAGE gel analysis of Cas12a-mediated trans-cleavage of FAM-labeled ssDNA in the absence and presence of target dsDNA. (a) dsDNA: *A. baumannii*; crRNA: *Ab*-ITS-crRNA (b) dsDNA: bla_{OXA-23};. crRNA: bla_{OXA-23}-crRNA1. The reactions were incubated at 37 °C for 1 h and analyzed by 20% denaturing urea-PAGE. "+" indicates the presence of the corresponding component, while "–" indicates its absence.

2.2 Optimization of mineral oil volume for evaporation prevention in LAMP reactions

Mineral oil was added to prevent evaporation during the LAMP reaction. When less than 10 μL of mineral oil was layered on top of the reaction mixture, liquid globules still formed on the caps of the 8-tube PCR strips, indicating its insufficient evaporation prevention. In contrast, both 15 μL and 20 μL of mineral oil effectively prevented evaporation (Figure S3). To ensure a successful reaction, 20 μL of mineral oil was used in the following experiments.

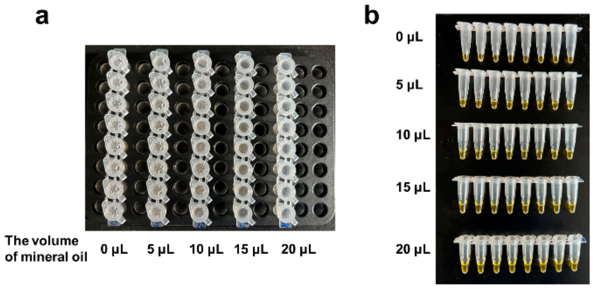

Figure S3. Effect of mineral oil volume on evaporation prevention in LAMP reactions. Different volumes of mineral oil (ranging from 10 μL to 20 μL) were layered on top of the LAMP reaction mixture. a. Top view of 8-tube PCR strips after incubation at 63 °C for 55 min. b. Side view of 8-tube PCR strips incubation at 63 °C for 55 min.

2.3. Optimization of ROX-labeled reporter probe in the one-pot LAMP-CRISPR/Cas12a assay targeting the *bla*$_{OXA-23}$ gene

The concentration of the ROX-labeled reporter probe was also optimized. The fluorescence signal intensities for different concentrations of ROX-labeled probes in the reaction system were presented in Figure S4. The fluorescence signal intensity of 1 μM ROX-labeled reporter probe added tests was higher than other tests, which was consistent with the results of the FAM-labeled probe. Therefore, 1 μM ROX-labeled reporter probe was added to the *bla*$_{OXA-23}$ assay in the following tests.

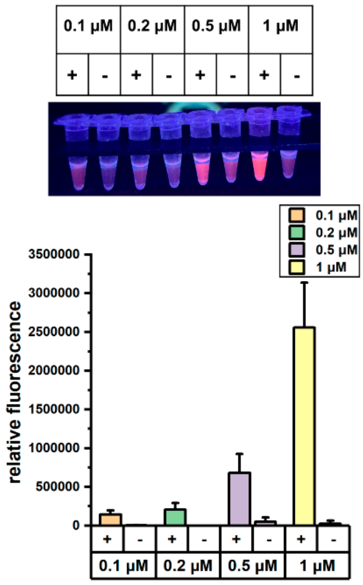

Figure S4. The fluorescence signals of LAMP-CRISPR/Cas12a assays with varying concentrations of ROX-labeled reporter probes.

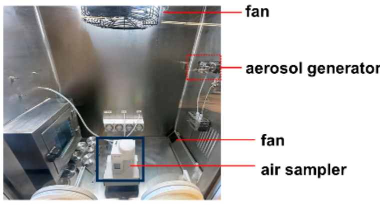

Figure S5. Simulation of aerosol sample collection.

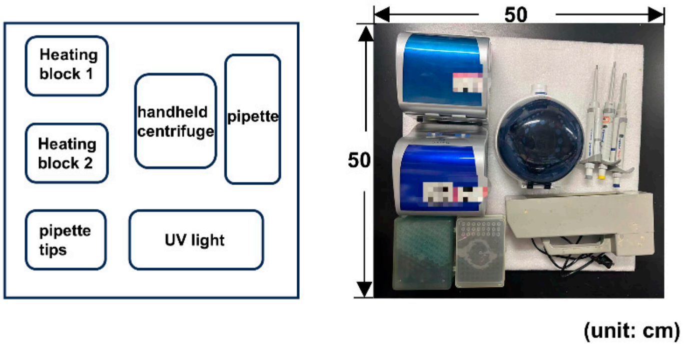

Figure S6. Minimum equipment required for the CLC platform. The essential equipment for running the CLC protocol (from DNA release to result readout) includes pipettes, pipette tips, a UV light source, a handheld centrifuge, and two heating blocks (one for 200 µL tubes and one for 1.5 mL centrifuge tubes). All components can be integrated into a 50 × 50 cm² suitcase for convenient on-site detection.

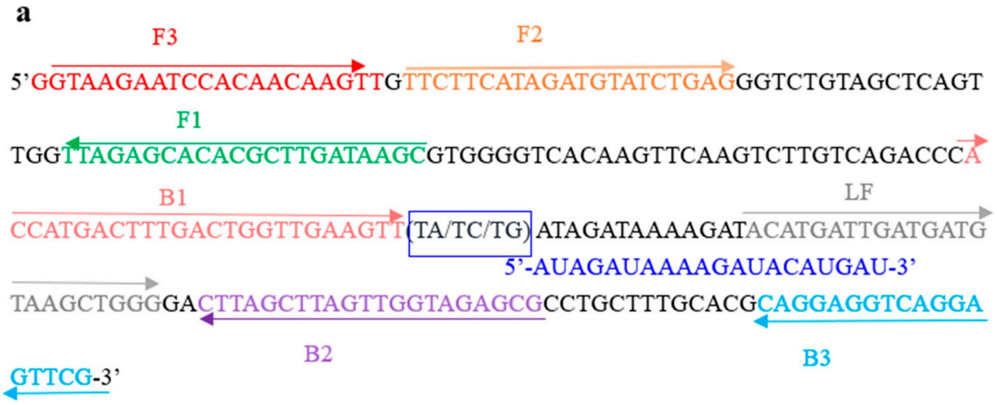

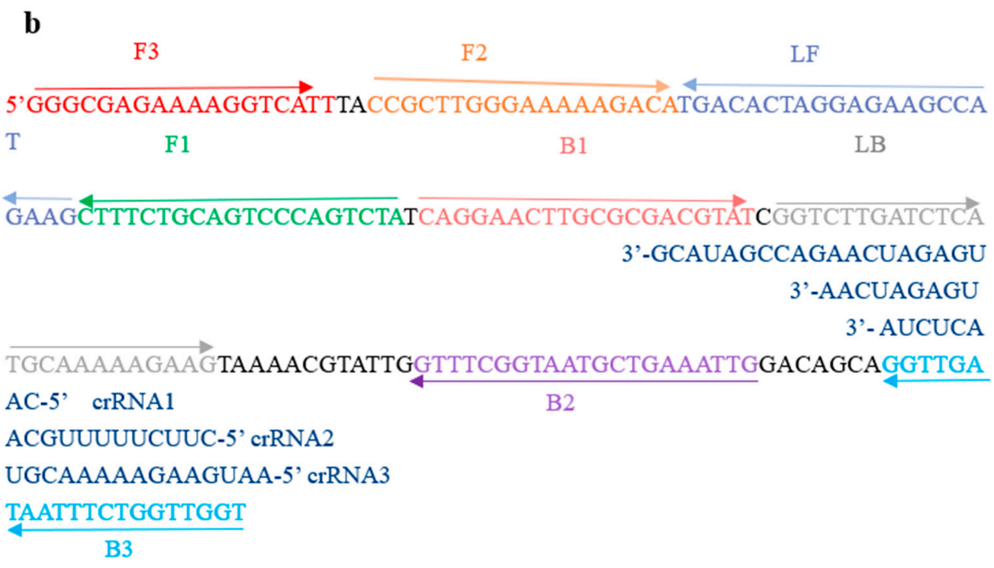

Figure S7. Primers and crRNAs design of the one pot LAMP-CRISPR/Cas12a assay. a. Part of nucleotide sequence of the *Ab*-ITS gene. The sequences in the blue rectangular were artificially added by the BIP primer. b. Part of nucleotide sequence of the *bla*$_{\text{OXA-23}}$ gene.

3. Supplementary table

Table S1. Comparison of multiple detection methods for *A. baumannii* and the *bla*$_{OXA-23}$ gene

Target	Sample type	Sample pretreatment	Technique	Readout method	Enzyme used	LOD	Time	Minimum professional equipment requirement	Ref
A. baumannii	Air sample	/	Culture	/	/	/	24 ~ 48 h	Incubator	[2]
	Spiked clinical swab and bronchoalveolar lavage fluid samples	/	Phage-based Taqman qPCR method	Fluorescence	DNA polymerase	10 CFU/mL	4 h	Real-time PCR system	[3]
		/	Taqman qPCR	Fluorescence	DNA polymerase	10^5 CFU/mL	~ 1 h	Real-time PCR system	[3]
	Bacterial culture	Boiling (10 min)	LAMP	Electrophoresis gel and visual turbidity assessment	Bst DNA polymerase	10 CFU/reaction (10 CFU/test)	> 79 min	Thermocycler (or water bath) and gel electrophoresis system	[4]
	Bacterial culture	/	Multiplex PCR	Electrophoresis gel	DNA polymerase	633 CFU/test	> 57 min	PCR machine and gel electrophoresis system	[5]
	Spiked blood Sample	Boiling (10 min)	Electrochemical genosensor	Electrochemistry	DNA polymerase; H_2O_2	10^3 CFU/mL	> 86 min	PCR machine and electrochemical Workstation	[6]
	Spiked fetal bovine serum samples	Microwave (1 min)	Protein-immobilized magnetic nanoparticle-based affinity approaches	Matrix-assisted laser/desorption ionization (MALDI)–MS	/	10^4 cells /mL or 10^5 cells /mL	~10 min	MALDI time-of-flight mass spectrometer	[7]
	Bacterial culture and simulated air samples	Chelex-100 based extraction method (5 min)	One-pot LAMP-CRISPR/Cas12a assay	Fluorescence and visual detection	Bst DNA polymerase; Cas12a	600 CFU/test (600 CFU/mL）	~ 60 min	Heating block and UV light	This study

Table S1 continued

Target	Sample type	Sample pretreatment	Technique	Readout method	Enzyme used	LOD	Time	Minimum professional equipment requirement	Ref
bla$_{OXA-23}$	Bacterial culture and clinical strains	Boiling (10 min)	Multiplex RPA-CRISPR/Cas12a (Two-step)	Fluorescence and visual detection	Recombinase; single-stranded binding protein; DNA polymerase; Cas12a	1.3 fg/μL of CRAb genomic DNA	50 min	A water bath, heating block, and a UV or blue light	[8]
	Bacterial culture and venous blood samples	Column-based extraction method	Droplet digital PCR	Fluorescence	DNA polymerase	300 fg/μL of CRAb genomic DNA	80 min	Droplet digital PCR system	[9]
	Bacterial culture and venous blood samples	Bacterial extraction kit	Quantitative PCR	Fluorescence	DNA polymerase	300 pg/μL of CRAb genomic DNA	~ 24 min	Real-time PCR system	[10]
	Sputum isolates	Commercial DNA extraction kit (15~30 min)	LAMP	Electrophoresis gel	Bst DNA polymerase	10 pg/μL of CRAb genomic DNA	> 40 min	Heating block and gel electrophoresis system	[11]
	Bacterial culture and simulated air samples	Chelex-100 (5 min)	One-pot LAMP-CRISPR/Cas12a assay	Fluorescence and visual detection	Bst DNA polymerase; Cas12a	174 fg/μL of CRAb genomic DNA	~ 60 min	Heating block and UV light	This study

Table S2. Oligonucleotides used in this study.

Oligonucleotides	Type	Sequence (5'-3')
Ab-ITS-LAMP	F3	GATTGGTAAGAATCCACAACA
	B3	CGAACTCCTGACCTCCTG
	FIP	GCTTATCAAGCGTGTGCTCTAACTTGTTCTTCATAGATGTATCTGAG
	BIP-WT	ACCATGACTTTGACTGGTTGAAGTTCGCTCTACCAACTAAGCTAAG
	BIP-TC	ACCATGACTTTGACTGGTTGAAGTTTCCGCTCTACCAACTAAGCTAAG
	BIP-TA	ACCATGACTTTGACTGGTTGAAGTTTACGCTCTACCAACTAAGCTAAG
	BIP-TG	ACCATGACTTTGACTGGTTGAAGTTTGCGCTCTACCAACTAAGCTAAG
	LF	ACATGATTGATGATGTAAGCTGGG
bla$_{\text{OXA-23}}$-LAMP	F3	GGGCGAGAAAAGGTCATT
	B3	ACCAACCAGAAATTATCAACC
	FIP	TAGACTGGGACTGCAGAAAGCCCGCTTGGGAAAAAGACA
	BIP	CAGGAACTTGCGCGACGTATCAATTTCAGCATTACCGAAAC
	LF	TCATGGCTTCTCCTAGTGTCA
	LB	GGTCTTGATCTCATGCAAAAAGAAG
FAM-labeled reporter probe	ssDNA molecule reporter	FAM-TTATTATT-BHQ-1
ROX-labeled reporter probe	ssDNA molecule reporter	ROX-TTATTATT-BHQ-2

Table S2 continued

Ab-ITS-crRNA	CRISPR RNA	GGUAAUUUCUACUAAGUGUAGAU AUAGAUAAAAGAUACAUGAU
bla$_{\text{OXA-23}}$-crRNA1	CRISPR RNA	GGGUAAUUUCUACUAAGUGUAGAU CAUGAGAUCAAGACCGAUACG
bla$_{\text{OXA-23}}$-crRNA2	CRISPR RNA	GGUAAUUUCUACUAAGUGUAGAU CUUCUUUUUGCAUGAGAUCAA
bla$_{\text{OXA-23}}$-crRNA3	CRISPR RNA	GGGUAAUUUCUACUAAGUGUAGAU AUCUCAUGCAAAAAGAAGUAA
bla$_{\text{OXA-23}}$-F	sequencing primer	CACTAGGAGAAGCCATGAAG
bla$_{\text{OXA-23}}$-R	sequencing primer	CAGCATTACCGAAACCAATACG
FAM-labeled ssDNA	ssDNA molecule	FAM-TTTTTTTATTATTT

References

1. Li, S.Y.; Cheng, Q.X.; Wang, J.M.; Li, X.Y.; Zhang, Z.L.; Gao, S.; Cao, R.B.; Zhao, G.P.; Wang, J. CRISPR-Cas12a-assisted nucleic acid detection. *Cell Discov* **2018**, *4*, 20, doi:10.1038/s41421-018-0028-z.
2. Wong, S.C.; Lam, G.K.M.; Chen, J.H.K.; Li, X.; Ip, F.T.F.; Yuen, L.L.H.; Chan, V.W.M.; Auyeung, C.H.Y.; So, S.Y.C.; Ho, P.L.; et al. Air dispersal of multidrug-resistant Acinetobacter baumannii: implications for nosocomial transmission during the COVID-19 pandemic. *Journal of Hospital Infection* **2021**, *116*, 78-86,

doi:10.1016/j.jhin.2021.08.005.

3. Luo, J.; Liu, M.; Wang, P.; Li, Q.; Luo, C.; Wei, H.; Hu, Y.; Yu, J. Evaluation of a direct phage DNA detection-based Taqman qPCR methodology for quantification of phage and its application in rapid ultrasensitive identification of Acinetobacter baumannii. *BMC Infect Dis* **2022**, *22*, 523, doi:10.1186/s12879-022-07493-1.

4. Esmaeeli, A.; Ravan, H.; Hassanshahian, M.; Khaleghi, M. Rapid LAMP-based detection of A. baumannii and aminoglycoside resistance genes in ESKAPE pathogens. *Microb Pathog* **2025**, *202*, 107436, doi:10.1016/j.micpath.2025.107436.

5. Chen, T.L.; Siu, L.K.; Wu, R.C.; Shaio, M.F.; Huang, L.Y.; Fung, C.P.; Lee, C.M.; Cho, W.L. Comparison of one-tube multiplex PCR, automated ribotyping and intergenic spacer (ITS) sequencing for rapid identification of Acinetobacter baumannii. *Clin Microbiol Infect* **2007**, *13*, 801-806, doi:10.1111/j.1469-0691.2007.01744.x.

6. Kanapathy, S.; Obande, G.A.; Chuah, C.; Shueb, R.H.; Yean, C.Y.; Banga Singh, K.K. Sequence-Specific Electrochemical Genosensor for Rapid Detection of bla(OXA-51-like) Gene in Acinetobacter baumannii. *Microorganisms* **2022**, *10*, doi:10.3390/microorganisms10071413.

7. Bai, Y.L.; Shahed-Al-Mahmud, M.; Selvaprakash, K.; Lin, N.T.; Chen, Y.C. Tail Fiber Protein-Immobilized Magnetic Nanoparticle-Based Affinity Approaches for Detection of Acinetobacter baumannii. *Anal Chem* **2019**, *91*, 10335-10342, doi:10.1021/acs.analchem.9b02964.

8. Zhou, Z.; Liang, L.; Liao, C.; Pan, L.; Wang, C.; Ma, J.; Yi, X.; Tan, M.; Li, X.; Wei, G. A multiplex RPA coupled with CRISPR-Cas12a system for rapid and cost-effective

identification of carbapenem-resistant Acinetobacter baumannii. *Front Microbiol* **2024**, *15*, 1359976, doi:10.3389/fmicb.2024.1359976.

9. Kou, X.; Zhu, D.; Zhang, Y.; Huang, L.; Liang, J.; Wu, Z.; Liu, Z.; Guan, C.; Yu, L. Development and clinical validation of a dual ddPCR assay for detecting carbapenem-resistant Acinetobacter baumannii in bloodstream infections. *Front Microbiol* **2024**, *15*, 1338395, doi:10.3389/fmicb.2024.1338395.

10. Yu, L.; Kou, X.; Liu, Z.; Guan, C.; Sun, B. Establishment and validation of a dual qPCR method for the detection of carbapenem-resistant Acinetobacter baumannii in bloodstream infections. *Front Cell Infect Microbiol* **2025**, *15*, 1490528, doi:10.3389/fcimb.2025.1490528.

11. Kim, H.J.; Kim, H.S.; Lee, J.M.; Yoon, S.S.; Yong, D. Rapid detection of Pseudomonas aeruginosa and Acinetobacter baumannii Harboring bla(VIM-2), bla(IMP-1) and bla(OXA-23) genes by using loop-mediated isothermal amplification methods. *Ann Lab Med* **2016**, *36*, 15-22, doi:10.3343/alm.2016.36.1.15.
